# Conformal Radiotherapy Facilitates the Delivery of Concurrent Chemotherapy and Radiotherapy: A Case of Primitive Neuroectodermal Tumour of the Chest Wall

**DOI:** 10.1080/13577140020008101

**Published:** 2000-09

**Authors:** C. E. Coles, N. Twyman, H. M. Earl, N. G. Burnet

**Affiliations:** Department of Oncology Box 193 Addenbrooke's Hospital Hills Road Cambridge CB2 2QQ UK

## Abstract

We illustrate the principle of conformal radiotherapy by discussing the case of a patient with a primitive neuroectodermal tumour of the chest wall. Recent advances in radiotherapy planning enable precise localization of the planning target volume (PTV) and normal organs at risk of irradiation. Customized blocks are subsequently designed to produce a treatment field that ‘conforms’ to the PTV. The use of conformal radiotherapy (CRT) in this case facilitated the delivery of concurrent chemotherapy and radiotherapy by significantly reducing the volume of red marrow irradiated.The lack of acute and late toxicities was attributed to optimal exclusion of normal tissues from the treatment field, made possible by CRT.


Sarcoma (2000) **4**, 129–133

PRACTICE POINT

### Conformal radiotherapy facilitates the delivery of concurrent chemotherapy and radiotherapy: a case of primitive neuroectodermal tumour of the chest wall

C. E. COLES, N. TWYMAN, H. M. EARL & N. G. BURNET

*Department of Oncology, Addenbrooke's Hospital, Cambridge, UK*

**Abstract**
We illustrate the principle of conformal radiotherapy by discussing the case of a patient with a primitive neuroectodermal tumour of the chest wall. Recent advances in radiotherapy planning enable precise localization of the planning target volume (PTV) and normal organs at risk of irradiation. Customized blocks are subsequently designed to produce a treatment field that 'conforms' to the PTV. The use of conformal radiotherapy (CRT) in this case facilitated the delivery of concurrent chemotherapy and radiotherapy by significantly reducing the volume of red marrow irradiated. The lack of acute and late toxicities was attributed to optimal exclusion of normal tissues from the treatment field, made possible by CRT.

**Key words:** *primitive neuroectodermal tumour of chest wall, conformal radiotherapy*

### Introduction

Conformal radiotherapy (CRT) is of proven benefit in reducing normal tissue toxicity without jeopardizing local control in carcinoma of prostate.[1] Similar considerations apply to many other sites since CRT allows accurate localization of the planning target volume with sparing of critical structures. Application of these modern radiotherapy techniques will improve outcome by reducing normal tissue complications and may increase local tumour control by allowing dose escalation. These concepts are also highly relevant in combined modality treatment using concurrent chemotherapy and radiotherapy. Advantages of concomitant treatment are avoidance of delay in both modalities, and possible greater tumour kill due to the sensitizing effects of chemoradiation. Disadvantages include increased radiotherapy side-effects as well as the effects of overlapping toxicities. An important example is irradiation of large volumes of bone marrow causing exacerbation of chemotherapy-induced myelosuppression, resulting in treatment delay and thus possibly compromising cure. We discuss a case in which CRT reduced the bone marrow volume irradiated and thus facilitated concurrent treatment.

### Case report

A 20-year-old farmer presented with a 2-week history of a painful mass on the left posterolateral chest wall. Closer questioning revealed he had had mild discomfort in this area for some months. There was no other relevant history and he was otherwise well. A computerized tomography (CT) scan of the thorax showed a 6-cm soft tissue mass surrounding and destroying the posterolateral left ninth rib. Tumour extended through the chest wall to involve serratus anterior and a small left pleural effusion was present. Biopsy showed small round blue cells in keeping with a primitive neuroectodermal tumour (PNET), and this was confirmed by immunohistochemistry.

He commenced etoposide, vincristine, adriamycin, ifosfamide and actinomycin D (EVAIA) chemotherapy with dosages and scheduling as per the high-risk arm of European Intergroup Cooperative Ewings Sarcoma Study (EICESS) 92 protocol (Table 1). Following five cycles of chemotherapy, surgery was undertaken. A central portion of the eighth rib and the entire ninth rib with the attached intercostal soft tissue were resected. There were some flimsy adhesions to the left lower lobe, but no direct involvement of the lung or the diaphragm. A single 1.5×1.0 mm tumour deposit was the only residual disease identified following chemotherapy. This was clear of the closest resection margin by 1.5 mm.

Radiotherapy was given at this stage as per the EICESS 92 protocol. There were no delays, as the planning and treatment programme was organized in advance to anticipate the waiting list. The patient was

Correspondence to: Dr C. E. Coles, Department of Oncology, Box 193, Addenbrooke's Hospital, Hills Road, Cambridge, CB2 2QQ, UK. Tel: 01223 274312; Fax: 01223 412213; E-mail: cam48@cam.ac.uk

1357-714Xprint/1369-1643online/00/030129-05 © 2000 Taylor & Francis Ltd
*DOI: 10.1080/13577140020008101*

**Table 1.** *EVAIA chemotherapy for high-risk patients as per EICESS 92 protocol*

| Drug | Dose (mg/m$^2$) | Day | Cycle |
|---|---|---|---|
| Etoposide | 150 | 1, 2, 3 | 3-weekly |
| Vincristine | 1.5 | 1 | 3-weekly |
| Adriamycin | 20 | 1, 2, 3 | 6-weekly, alternating with actinomycin D |
| Ifosfamide | 2000 | 1, 2, 3 | 3-weekly |
| Actinomycin D | 0.5 | 1, 2, 3 | 6 weekly, alternating with adriamycin |
| Mesna | 2000 | 1, 2, 3, 4 | 3-weekly |

positioned in a customized vacuum bag to ensure reproducible set-up, and his arms were raised above his head to permit CT scanning. The phase 1 target volume encompassed the entire left hemithorax including the tumour, and phase 2 covered the original tumour site with a margin. Prior chemotherapy and surgery had removed the gross tumour volume and therefore the prosthesis was taken to represent the clinical target volume (CTV) in phase 2. A margin of 1 cm around the CTV was allowed for set-up error and formed the planning target volume (PTV). Accurate lung compensation was possible using the information from the CT scan.

Conformal planning for phase 1 permitted exclusion of the whole of the inferior and superior vertebral bodies and parts of all the others, thus sparing a substantial volume of red marrow. Fig. 1 shows the axial view, regions of interest and beam's eye view of the open field arrangement without blocks. The same views are shown in Fig. 2, but the addition of conformal blocks illustrates the vertebral shielding. The humoral head and ipsilateral kidney were also shielded. Addenbrooke's radiotherapy planning system (ARPS) was used to create dose-volume histograms (DVHs) of the vertebrae both before and after shielding. Without conformal blocks, the volume of the spine receiving more than 10% of the tumour dose (1.95 Gy) was 27.9 mm$^3$; with shielding, this was reduced by 50% to 13.95 cm$^3$. The DVHs are shown in Fig. 3. Phase 2 was planned conformally to limit the volume of lung and ribcage receiving the higher dose. Movement of the chest wall was screened in the simulator to ensure adequate coverage of the target volume throughout respiration. This demonstrated very little movement at the original tumour site due to resection and repair with a bone cement prosthesis.

The patient received a blood transfusion prior to radiotherapy and his haemoglobin was maintained above 12 g/dl. This is standard policy in our Sarcoma Unit as this has been shown to influence oxygenation and tumour response.[2] Phase 1 consisted of 19.5 Gy lung dose in 13 fractions over 2.5 weeks using parallel opposed anterior and posterior fields. Phase 2 consisted of 36 Gy tumour dose in 20 fractions over 4 weeks using a three-field plan. The photon energy was 8 MV for both phases. During treatment of the hemithorax, chemotherapy was stopped to avoid potential pulmonary sensitization leading to lung damage. Chemotherapy was resumed during phase 2, but adriamycin and actinomycin D were omitted. Concomitant chemoradiation was well tolerated with the only side-effect being mild fatigue.

A total of 13 courses of EVAIA chemotherapy were administered. Radiotherapy was given concurrently with cycles 9 and 10 of chemotherapy. There was only one delay due to neutropenic sepsis, which preceded radiotherapy treatment. Small dose reductions of 10–20% were made during the last five cycles due to persisting neutropenia. This intensive chemotherapy regimen often requires dose reductions or treatment delays, even in the absence of concurrent radiotherapy.

### Discussion

PNET tumours of the chest wall are uncommon, but we have experience of two other cases that were treated in a similar way. All had minimal acute toxicity and no long-term treatment-related sequelae to date. Median length of follow-up was 28 months, with a range of 16–43 months. This suggests that CRT confers considerable advantage by decreasing late normal tissue damage with no loss of local control. In soft tissue sarcomas specifically, it has been shown that advanced radiotherapy planning can reduce the volume of normal tissue irradiated by around 30%.[3]

Chemotherapy dose intensification has been carried out over the last 10–20 years, and this has led to increasing acute toxicity, particularly with haematological and infective complications.[4] In the recently published results from the UK study for axial primary tumours, the combination of surgery and radiotherapy, as well as chemotherapy, seemed to be more effective than surgery alone. There were no local relapses amongst 11 patients receiving radiotherapy compared with 12 out of 41 (29%) of patients relapsing where radiotherapy was not given. The local control rates in this study were similar to the Cooperative Ewings Sarcoma Study (CESS) 86 study.[4] The concept of reduced toxicity with radiation technique is extremely important, as it is highly likely that future chemotherapy regimens will include dose intensification and high-dose treatments. For example, decreasing the amount of marrow involved in the radiotherapy field will facilitate the delivery of planned chemotherapy dose with no additional delays.

The technique of CRT planning involves outlining

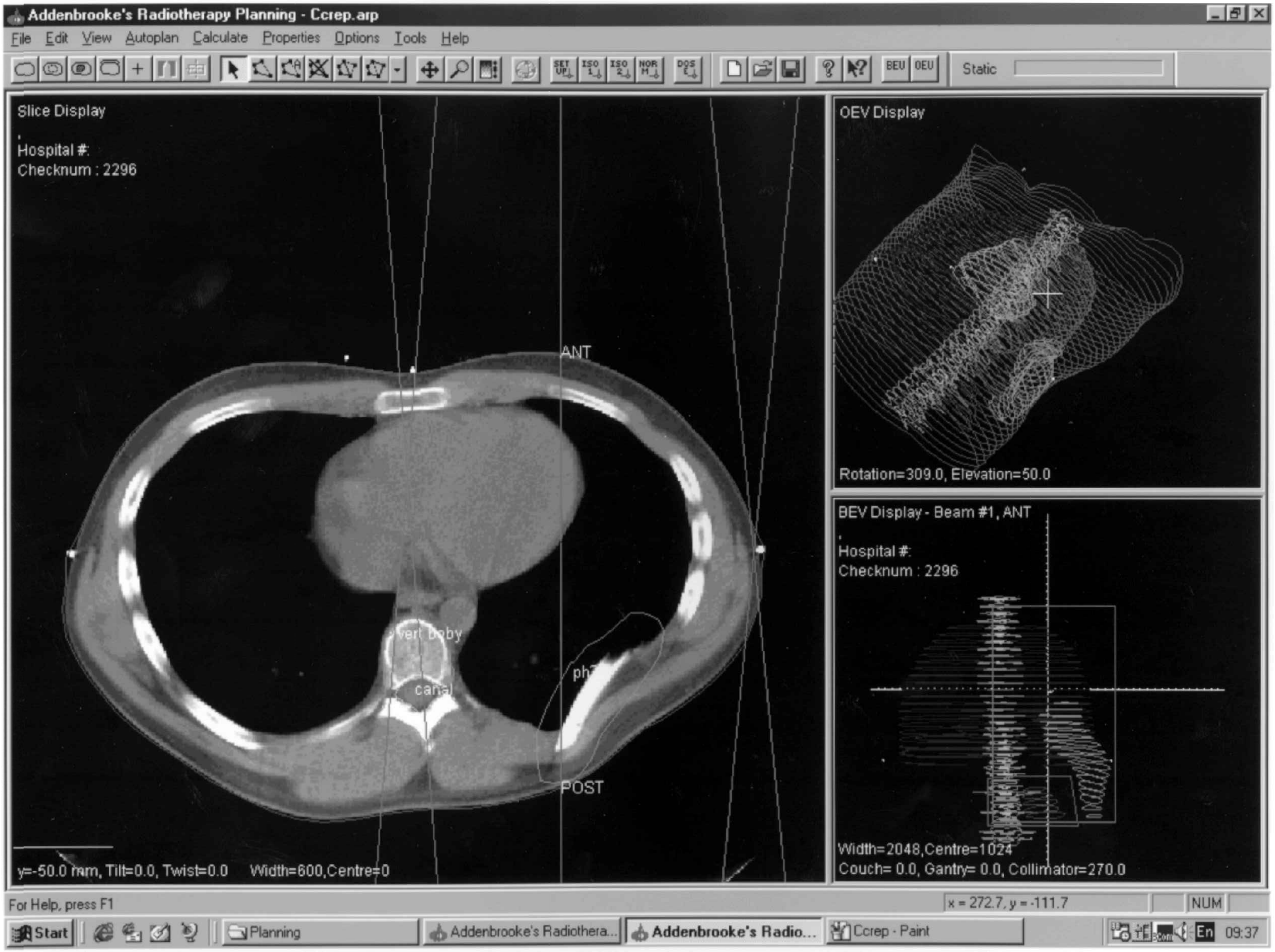

**Figure 1**. *Open radiotherapy field arrangement. Main picture: axial view showing position of open field. Top right: operator's eye view showing all regions of interest. Bottom right: beam's eye view of open fields with kidney block.*

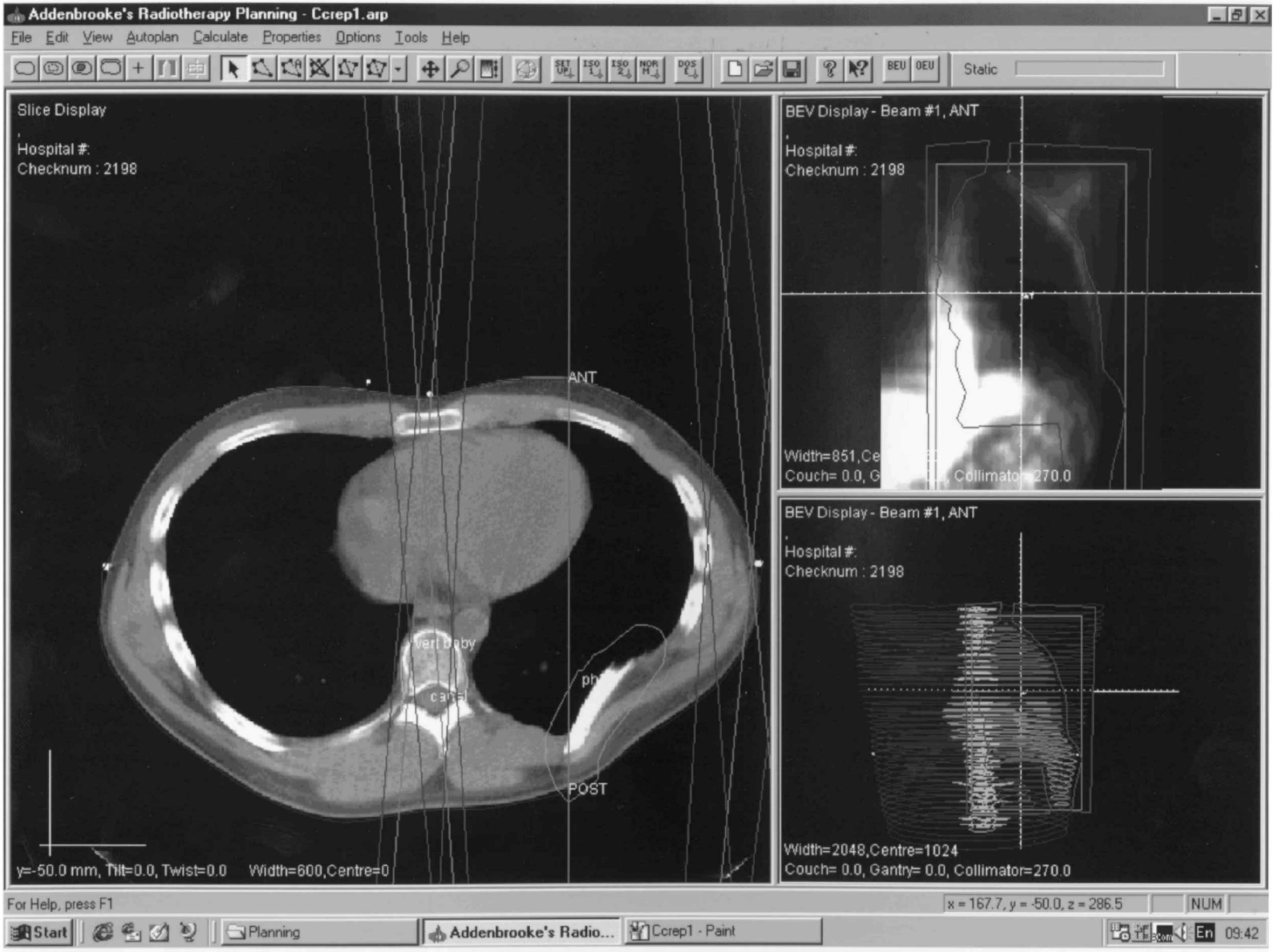

**Figure 2**. *Field arrangement with conformal blocks. Main picture: axial view showing open fields and conformal blocks. Top right: beam's eye view of conformal blocks and digitally reconstructed radiographs. Bottom right: beam's eye view of conformal blocks showing all regions of interest.*

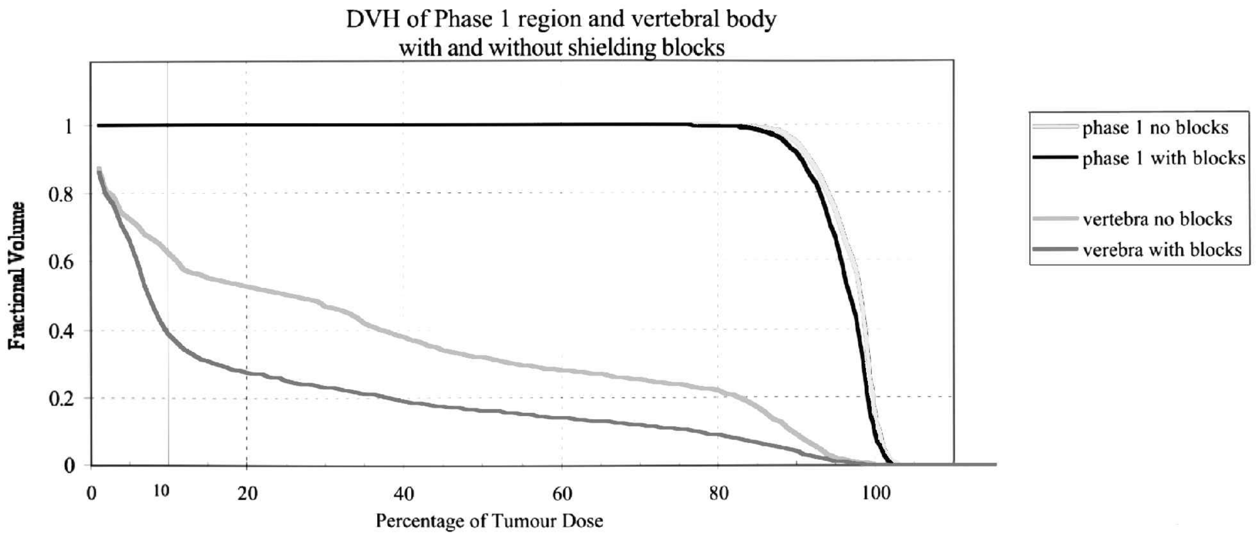

**Figure 3**. *DVH of phase 1 region and vertebral bodies with and without shielding blocks.*

the axial CTV on each CT slice. This can be time-consuming, given that the superior–inferior CTV dimensions are often large. We have developed ARPS, an advanced planning system, with algorithms to 'grow' the CTV isotropically into the PTV. This software has reduced clinician-planning time and greatly assisted verification due high-quality digitally reconstructed radiographs.

Hemithoracic radiotherapy has not been definitely proven to be necessary in patients with PNET of the chest wall and pleural effusions. The best data, however, come from the CESS group. These show an overall survival of 60% and an event-free survival of 50% at 5 years, using their routine strategy to irradiate the hemithorax in these patients.[5] Quality of radiotherapy planning has been shown to be important in patients with Ewing's tumour. The CESS 81 and 86 studies demonstrated that better planning conferred an improved outcome; CRT is likely to extend this advantage.[6]

Previous studies have addressed the issue of dose in Ewing's and PNET tumours. A dose less than 40 Gy is ineffective, but in the dose range of 40–66 Gy there is little evidence of a dose–response relationship.[7] This data set included all tumour sites, and volume data were not available. Where volume has been measured prospectively in studies, it has been shown to be an important prognostic factor.[6] In large tumours, e.g. more than 100 ml,[6] it would be expected that a higher dose would improve local control. This could be achieved with acceptable acute toxicity using CRT. Reduction of the volume of normal tissue treated will also reduce late normal tissue effects and possibly decrease the incidence of radiation-induced second malignancies.

The combined approach to this condition, using chemotherapy, surgery and radiotherapy, is leading to improvements in local control and survival. In these circumstances, the issue of late normal tissue effects becomes all the more important.

We presented this case as an illustration of the application of standard CRT techniques in patients with PNET of the chest wall. CRT, within the multidisciplinary setting, is of value for these patients, most of whom are young and with an otherwise long life expectancy.

### References

1. Dearnaley DP, Khoo VS, Norman AR, Meyer L, Nahum A, Tait D, Yarnold J. Comparison of radiation side-effects of conformal and conventional radiotherapy in prostate cancer: a randomised trial. *Lancet* 1999;353 (9149):267–72.
2. Bush RS. The significance of anemia in clinical radiation therapy. *Int J Radiat Oncol Biol Phys* 1996;12:2047–50.
3. Robinson MH, Bidmead AM, Harmer CL. Value of conformal planning in the radiotherapy of soft tissue sarcoma. *Clin Oncol* 1992;4:290–3.
4. Craft A, Cotterill S, Malcolm A, Spooner D, Grimer R, Souhami R, Imeson J, Lewis I. Ifosfamide-containing chemotherapy in Ewing's sarcoma: the Second United Kingdom Children's Cancer Study Group and the Medical Research Council Ewing's Tumor Study. *J Clin Oncol* 1998;16:3628–33.
5. Schuck A, Hofmann J, Rube C, Hillmann A, Ahrens S, Paulussen M, Jurgens H, Dunst J, Willich N. Radiotherapy in Ewing's sarcoma and PNET of the chest wall: results of the trials CESS 81, CESS 86 and EICESS 92. *Int J Radiat Oncol Biol Phys* 1998;42:1001–6.
6. Dunst J, Jürgens H, Sauer R, Pappe H, Paulussen M, Winkelman W, Rübe C. Radiation therapy in Ewing's sarcoma: an update of the CESS 86 trial. *Int J Radiat Oncol Biol Phys* 1995;32:919–30.
7. Burnet NG, Bliss JM, Harmer CL. The impact of radiotherapy dose on local control of Ewing's sarcoma of bone. *Sarcoma* 1997;1:31–8.
